# The Institutional Worker

**Published:** 1918-04-13

**Authors:** 


THE
### INSTITUTIONAL WORKER
**Being a Special Supplement to "The Hospital"**

### OUR BUREAU OF INFORMATION.

**Rules for Correspondents.**

1. Every letter must be accompanied by the coupon to be cut from the back cover (inside page) of The Hospital, current issue, and must contain the name and address of the correspondent with pseudonym for publication if desired. Replies by post cannot be given save under exceptional circumstances at the Editor's discretion.

2. Letters from Approved Homes in reply to special needs published in the Bureau should state terms and full particulars, and be sent prepaid under cover to the Editor of the Bureau with name written across coupon for identification.

3. Proprietors of Homes which have not yet been entered on the List of Approved Homes, but have spare accommodation likely to suit special needs, are invited to write for an application form for registration. The fee for registration, which includes two announcements of the Home in the Bureau and other privileges, is 10s.

4. All communications to be addressed to the Editor of The Hospital, 28 Southampton Street, Strand, London, W.C. 2, and marked "Bureau of Information."

### INSTITUTION FACTS AND FIGURES.

### How to Sterilise Rubber Gloves.

**Answer to February Question.**

**By "O. T."**

The question was:

**Describe the best method of sterilising rubber gloves, to be used during operations, to ensure their being perfectly sterilised with the minimum amount of damage to the rubber.**

There are several methods of sterilising rubber gloves:—

1. *Boiling in a Steriliser.*—In this case it is advisable to bring the water to boiling-point first. The gloves are immediately immersed at that temperature, and are allowed to remain in the water and kept boiling for about fifteen minutes. Carbolic acid 1 per cent. may be added, but not necessarily. The gloves are then removed, placed on a sterile towel to dry, and finally rubbed over with talc powder ready for use.

2. *Wet Heat.*—Thoroughly cleanse the gloves with soap and water, carefully removing all powder, wrap in towel or napkin, and place in steam steriliser.

3. *Submersion in Chemical Germicides.*— Submerge the gloves after cleansing in any antiseptic solution, as bichloride, carbolic, Lysol, formalin, etc., of the usual strength. These antiseptics do not affect the rubber.

4. *Formalin Fumes.*—Place gloves into a "Shering" oven and burn one or two five-grain formalin pastilles. Allow the gloves to remain in the oven for ten minutes.

5. *Sulphuric Acid.*—The gloves are washed in running water and dried. They are then placed in a 5 per 1,000 solution of sulphuric acid, and left for ten or twelve hours, after which they are rinsed in salt solution, and are ready for use.

6. *Mercuric Cyanide.*—An 8-oz. bottle is half filled with powdered talc, to which has been added two tablets of mercuric cyanide, when the bottle is filled with alcohol, 90 per cent. This liquid is kept for use as required. The gloves, before being treated, are first washed in hot water, rinsed, placed in a sterile dish, and the well-shaken liquid poured over them. They are then removed and placed on a sterile towel to dry. The alcohol upon evaporation leaves the gloves completely covered with the dry talc-cyanide powder ready for use. Absolute safety is claimed for rubber gloves treated in this way.

General Precautions.

(*a*) Gloves should at all times be removed from boiler, antiseptic solutions, or oven by means of sterile gauze or forceps. This is an essential precaution.

(*b*) *Drawing On and Removing Gloves from Hands.*—Rub the gloves over with talc powder or lubricate with sterile glycerin or soap-suds. Grasp the gauntlet with a sterile cloth, insert hand, and gently draw on. The same precaution should be observed in removing gloves—*i.e.*, grasp the gauntlet and draw over the hand. Do not attempt to remove by pulling ends of fingers. If a glove finger should be accidentally cut or punctured, roll a tissue cot which has been sterilised over the damaged finger.

By observation of minor details in the keeping and handling of rubber gloves a large number may be saved, thereby reducing this item of expenditure to a very low point.

### Hot Dinner-Plates.

**Answer to February Question.**

**By "Patricia."**

The question was:

**A hospital has four wards, each containing twenty beds. The dinners are served from one inadequate general kitchen and there is no arrangement for heating the plates. Each ward has a very tiny kitchen, which holds a small gas-ring. How can the plates be best heated?**

In a hospital where there is not sufficient accommodation in the general kitchen for heating the plates for patients' dinners, and in which there is only a small gas-ring in each ward kitchen, the plates can be got beautifully hot in the ward kitchens by putting them into a deep bowl and pouring boiling water over them about ten minutes before they are required, taking care that all the plates are covered with the boiling water. The bowl should then be covered until the dinners arrive.

When the sister is ready to serve dinners a nurse should take one plate at a time out of the hot water, wipe it with a dry towel, and hand it to the sister as she requires it.

If the nurse should find the water too hot for her hands, she can easily slip a tablespoon under each plate and raise it sufficiently out of the water to grasp it with a towel.

The plates for the nursing staff can be heated in the same way, only they must all be dried quickly just before being taken into the dining-room.

### APRIL QUESTION.

**A Register of Nurses' Ward Work.**

How should a book be ruled to give at a glance a record of nurses' different duties in male, female, and children's wards, and time spent at classes, etc.? The hospital is a training-school, and nurses take day and night duty. There are three floors for male patients, two for female patients, and a children's block.

### RULES.

The following rules must be observed :—

1. Contributions must be written on one side of the paper. Brevity and terseness are desirable features. The MSS. must bear the name and address of the sender and be accompanied by coupon to be cut from the back cover (inside page) of the current issue of The Hospital. A pseudonym must be chosen if the name is not to be published.

2. Contributions must be addressed to the Editor of The Hospital, Institutional Worker Supplement, 28 & 29 Southampton Street, Strand, London, W.C. 2, should reach him before the end of the current month, and be marked in the left-hand corner "Facts and Figures."

**A minimum payment of Five Shillings will be made for each published answer.**

### ENQUIRIES AND ANSWERS.

### *WAR MATTERS.*

**"War" Hospitals in Ireland.**

Yes; there are "war" hospitals in Ireland. Possibly you might obtain a post through the British Red Cross Society, 83 Pall Mall, S.W., or you could reply to advertisements for such positions or advertise your need in the nursing journals.—Colleen.

**Staff Nurse Q.A.I.M.N.S.R.**

We regret that we are unable to answer your inquiry as you have not given your name and address. Replies can be given in this column under a pseudonym, but it is essential that the name and address are given with each inquiry.

**Appointments in War Hospital.**

We cannot undertake to give the names of war hospitals requiring probationers or assistant nurses; the only course feasible or possible is to obtain such a post through the ordinary channels. See reply to "Colleen" above.—Nurse C. L. B.

### *ACKNOWLEDGMENTS.*

Hopeful.—We have made an announcement of your need in these columns in the hope that some of our Approved Homes will make an offer, and we will forward to you any communications we may receive.

### *THE SICK AND IN NEED.*

**Home for Invalid Lady.**

There is a Home for invalid ladies of limited means at 36 Aubert Park, Highbury, and 1, 2, and 3 Highbury Terrace, London, N. A weekly payment of 15s. is required, payable quarterly in advance. There is, of course, a possibility of this charge being increased owing to present war conditions. If you could state exactly what could be paid and in what neighbourhood a Home is required, we would endeavour to ascertain if any of our Approved Homes could take the lady. —Mr. T. W.

**Home for Aged Lady Required.**

Could any of our Approved Homes either in the neighbourhood of Wolverhampton or London receive a lady seventy-seven years of age? She has for the past twelve months kept to her bed more or less, although, apart from the infirmities of old age, she is in fairly good health. She could pay probably from 30s. to £2 a week. Replies should be addressed "Hopeful," care of the Editor.

**The Editor will be glad to receive correspondence and to consider contributions upon all subjects relating to institutional work which affect the welfare of institutional workers.**

